# Macrolets: Outsized Extracellular Vesicles Released from Lipopolysaccharide-Stimulated Macrophages that Trap and Kill *Escherichia coli*

**DOI:** 10.1016/j.isci.2020.101135

**Published:** 2020-05-05

**Authors:** Wei Ding, Olivia C. Rivera, Shannon L. Kelleher, David I. Soybel

**Affiliations:** 1 Department of Surgery, Penn State College of Medicine and Milton S. Hershey Medical Center, Room# C4810, H149, 500 University Drive, Hershey, PA 17033, USA; 2 Department of Cellular & Molecular Physiology, Penn State Hershey College of Medicine, Hershey, PA 17033, USA; 3 Department of Biomedical & Nutritional Sciences, Zuckerberg College of Health Sciences, University of Massachusetts Lowell, Lowell, MA 01852, USA

**Keywords:** Biological Sciences, Immunology, Immune Response, Cell Biology, Specialized Functions of Cells

## Abstract

Macrophages release a variety of extracellular vesicles (EVs). Here we describe a previously unreported class of EVs that are released from macrophages in response to *Escherichia coli* endotoxin, lipopolysaccharide (LPS), that we have named "macrolets" since they are extruded as large "drop*lets*" released from *macro*phages. Morphologically, macrolets are anuclear, bounded by a single lipid membrane and structurally dependent on an actin cytoskeleton. Macrolets are enriched in tetraspanins and separable on this basis from their parent macrophages. Macrolets are distinguished from classic exosomes by their larger size (10–30 μm), discoid shape, and the presence of organelles. Macrolets are rich in both interleukin 6 (IL-6) and interleukin 6 receptor (IL-6R),and are capable of trapping and killing *E*. *coli* in association with production of reactive oxygen species. Our observations offer insights into the mechanisms by which macrophage activities may be amplified in sites of infection, inflammation, and healing.

## Highlights

• Macrolets, outsized extracellular vesicles, release from LPS-stimulated macrophages
• Macrolets are rich in tetraspanin proteins such as CD81, CD63, and CD9
• Macrolets capture and internalize *E. coli* bacteria within acidic compartments
• Macrolets kill *E. coli* by a mechanism associated with production of ROS and superoxide

## Graphical Abstract

**Figure:**
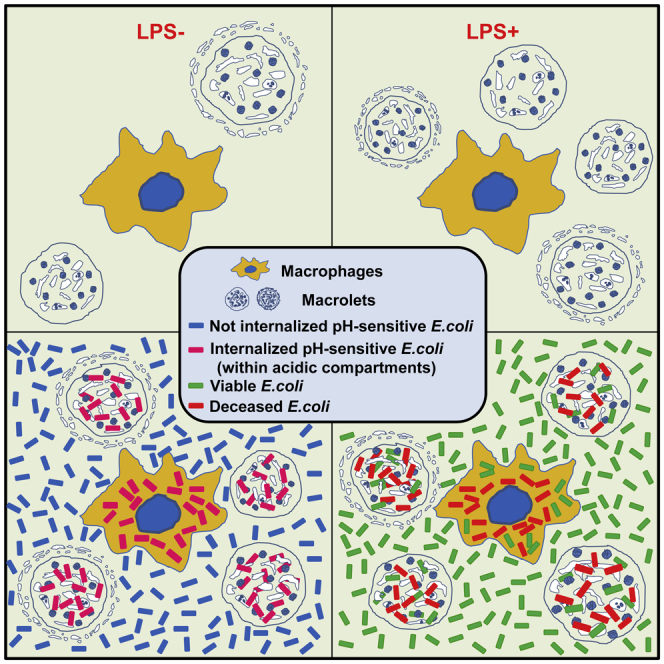

## Introduction

Macrophages play a pivotal role in the initiation, resolution, and persistence of inflammation (Dalli and Serhan, 2017, Ginhoux and Jung, 2014, Varol et al., 2015). Well-documented functions of macrophages include the production and secretion of cytokines and chemokines, phagocytosis of pathogens or dead/dying cells, and elaboration of extracellular matrix metalloproteinases (Ariel and Serhan, 2012, Hundertmark et al., 2018, Tomlin and Piccinini, 2018, Wight et al., 2017, Wynn and Barron, 2010). In response to danger signals such as *E*. *coli* endotoxin (lipopolysaccharide, LPS), macrophages lay extracellular traps (Doster et al., 2018, Sharma et al., 2017) and produce extracellular vesicles (EVs) containing a variety bioactive molecules (e.g., proteins, carbohydrates, lipids, and nucleic acids) that can influence local inflammatory responses in tissue and lead to phenotypic change in target cells (Esser et al., 2010, Ismail et al., 2013, O'Neill and Quah, 2008). These observations provide evidence for a wide variety of mechanisms by which macrophages are able to sense changes in their surrounding microenvironment and have their signature functions amplified and coordinated.

With respect to EVs, four categories have been reported, including exosomes, microvesicles or microparticles, apoptotic bodies, and oncosomes (Akers et al., 2013, Dreyer and Baur, 2016). All but oncosomes are produced by macrophages and are emerging as potentially consequential mediators in communications between macrophages and other cell types (Lanyu and Feilong, 2019, Zhu et al., 2017). EVs produced by macrophages are small relative to the diameter of their parent cells, with sizes ranging from 50 to 100 nm for exosomes (Bhatnagar et al., 2007), 200 to 1,000 nm for microvesicles (Ismail et al., 2013), and 1,000 to 5,000 nm for apoptotic bodies (Zhu et al., 2017). Recent studies, however, also provided evidence that EV classes of larger dimensions may also be released by human primary monocyte-derived dendritic cells (Kowal et al., 2016), with separation based on their enriched expression of tetraspanins such as CD63, CD81, or CD9. More recently, it has been shown that malignant cells can produce larger EVs or oncosomes (1–10 μm) that have an organized cytoskeleton and contain organelles (Johnson et al., 2017) or, in some cases, can be detected in the circulation of patients with cancer (Vagner et al., 2018). These findings suggest that larger classes of EVs might be produced by other cell types, including the macrophage.

Here we report a class of large EVs (10–30 μm) produced by human and mouse macrophage cell lines and primary human monocytes transformed to macrophages *ex vivo* and released in response to stimulation by LPS. We have named these EVs "macrolets," since, as demonstrated below, they appear as large drop***lets*** released from "***macro***phages". Macrolets are distinguished from currently known extracellular traps and EVs based on their size, morphology, contents, and apparent mode of biogenesis. As has been reported for exosomes (Kowal et al., 2016), macrolets are enriched in tetraspanins CD63, CD81, and CD9; have an organized cytoskeletal structure, and contain organelles such as mitochondria, lysosomes, and secretory compartments. In response to LPS stimulation, macrolets produce cytokines such as interleukin-6 (IL-6) as well as interleukin-6 receptor (IL-6R). Moreover, we show that macrolets are capable of trapping microbes such as *E*. *coli*, in association with classic bactericidal functions such as vesicular acidification and production of reactive oxygen species. These findings offer insights into novel and potentially multifunctional vehicles by which individual macrophages may communicate with other cells and amplify its danger signals within a space of inflammation and healing.

## Results

### Macrolets: Outsized Extracellular Vesicles Released by Macrophages in Response to *E*. *coli* Endotoxin (LPS)

**Figure 1:**
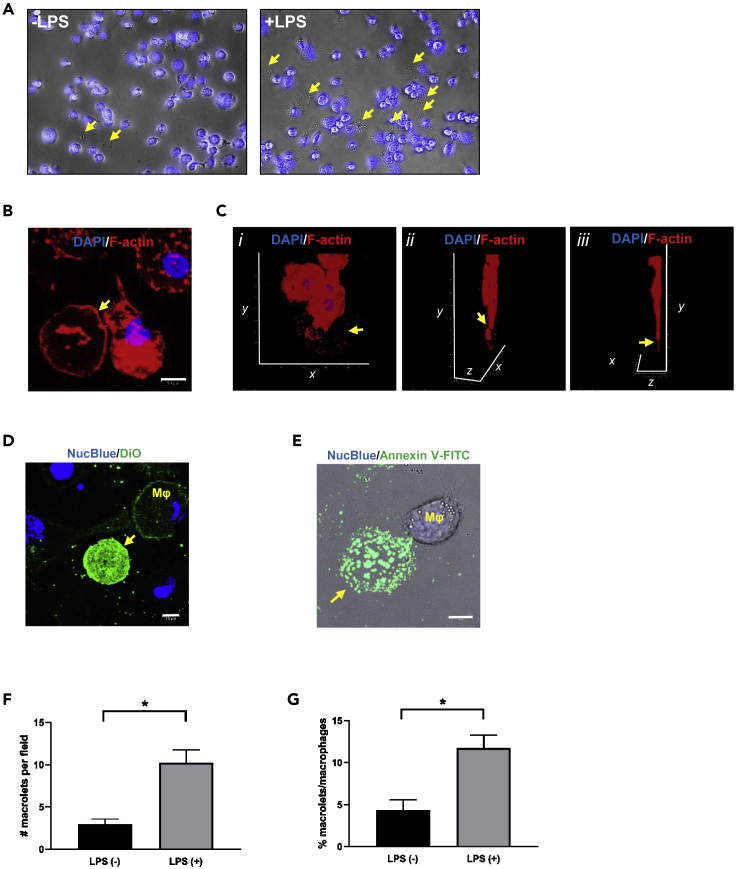
Heterogeneous Macrolets Are Released from THP-1 Macrophages upon *E*. *coli* Endotoxin LPS Stimulation (A) Representative images of THP-1 cells treated with lipopolysaccharide (+LPS; 100 ng/mL) or without (-LPS) for 4 h and stained with DAPI (40×⋅magnification). Images illustrate numerous anuclear particles (DAPI negative; arrow) are found in the extracellular space surrounding THP-1 macrophages. (B) Representative image of macrolets (arrow) stained with Alexa Fluor 594-phalloidin (red) illustrating that macrolets have an actin cytoskeleton. Scale bar, 10 μm. (Ci–iii) Z stack confocal images show that macrolet (arrow) stained with phalloidin (red) and DAPI (blue) is a discoid structure with the thickness of 1–1.5 μm. (D) Representative images of DiO (green) staining, which predominantly localizes to the cell membrane of some macrophages (Mφ). The macrolet (arrow) has an intense and diffuse DiO labeling. Scale bar, 10 μm. (E) Representative image showing a macrolet (arrow) has positive Annexin V-FITC (green) staining, but the parent macrophage (Mφ) that produced this macrolet has negative Annexin V-FITC labeling; multiple images (>20) were collected.Scale bar, 10 μm. (F) Data represent mean number of macrolets/field ±SE, quantified in four randomly selected images/treatment; ∗p < 0.01. (G) Data represent mean percent of macrolets/macrophages ±SE, quantified in four randomly selected images/treatment; ∗p < 0.01.

Under light microscopy, in both static images and in time-lapse imaging, we observed release of large EVs from human THP-1 macrophages after exposure to LPS (100 ng/mL, 4 h) as shown in Figure 1A. They did not contain nuclei (i.e., were DAPI-negative). As shown in time-lapse recordings (Videos S1A and S1B) they first appeared as hyperdense droplets forming out of membrane and cytoplasm and, once extruded, rapidly expanded to form discoid particles. Based on these initial observations we named these outsized EVs "macrolets," large cell drop*lets* released from *macro*phages.

Video S1A. Macrolets are Released from THP-1 Macrophages upon LPS Stimulation, Related to Figure 1 (A) and (B) Time-lapse images show that macrolets (highlighted with x1 in red) are budding off from LPS-stimulated parental THP-1 macrophages and released to the extracellular space.

Video S1B. Macrolets are Released from THP-1 Macrophages upon LPS Stimulation, Related to Figure 1 (A) and (B) Time-lapse images show that macrolets (highlighted with x1 in red) are budding off from LPS-stimulated parental THP-1 macrophages and released to the extracellular space.

Staining with phalloidin (Figure 1B) indicated that macrolets have an actin cytoskeleton providing structure both at the borders and in the interior. Under these conditions, macrolets were flattened, discoid structures (Figure 1C and Video S2), with thickness of a few micrometers and diameters ranging from 10 to 30 μm. Internal cytoskeletal organization was variable: a large proportion of macrolets have a well-organized cytoskeletal structure at their boundaries with relatively sparse interior staining (Figure S1A), whereas some macrolets had an outer "shell" and an interior "core" with somewhat fragmented staining (Figure S1B). In addition, macrolets were bounded by intact membrane: as shown in Figure 1D, the sides of the thin, discoid macrolet stain diffusely with the fluorescent, membrane-lipid reporter DiO’; DiOC_18_(3) (3, 3′-Dioctadecyloxacarbocyanine Perchlorate) (Vemula et al., 2014). Moreover, we found that macrolets are also rich in Annexin V (Figure 1E and Video S3). Annexin V is a Ca2^+^-dependent phospholipid-binding protein with high affinity for phospholipid phosphatidylserine (PS). Consistent with studies characterizing other EV proteins, Annexin V has been utilized for identification and purification of exosomes and microvesicles (Fitzner et al., 2011, Garzetti et al., 2014, Kowal et al., 2016, Pieters et al., 2019, Thery et al., 2018). Lastly, formation of macrolets was observed much less frequently in the absence of pre-treatment with LPS (Figures 1F and 1G).

Video S2. Macrolets Are Discoid in Shape and Are Rich in F-Actin Microfilaments, Related to Figure 1 Z stack confocal microscopic video shows that an anuclear (DAPI-) macrolet is a flat and discoid outsized vesicle rich in F-actin microfilaments (phalloidin; red).

Video S3. Macrolets Erupted from Their Parent Macrophages Are Rich in Annexin V, Related to Figure 1 Z stack confocal microscopic video shows that an anuclear (NucBlue-) macrolet erupted and released from its parent macrophage (stained with phalloidin; red) is rich in Annexin V (green).

To confirm macrolets were not a product of cell death, we induced apoptosis in THP-1 cells with etoposide (50 μM) for 4 h (Zhuang et al., 1998) and pyroptosis with a combination of LPS (100 ng/mL) and Nigericin (10 μM) (Bergsbaken et al., 2009, Broz, 2015, Cullen et al., 2015). As shown in Figure S2A, apoptosis was characterized by nuclear fragmentation and disruption of the cytoskeleton, which was distinct from anuclear macrolets with intact cytoskeletal structure. As shown in Figure S2B, pyroptosis was characterized by caspase-1 activation and associated with membrane and cytoskeletal disruption but no macrolets were detected as seen in cells treated with LPS alone (Figure S2C). In addition, we verified that the macrolets are produced by viable parent macrophages showing both Annexin V and PI negative staining (Figure S2D). These studies confirm that formation of the macrolets is an active process and not a by-product of two key pathways that lead to cell death.

### Organelles and Membrane Markers of Macrolets

**Figure 2:**
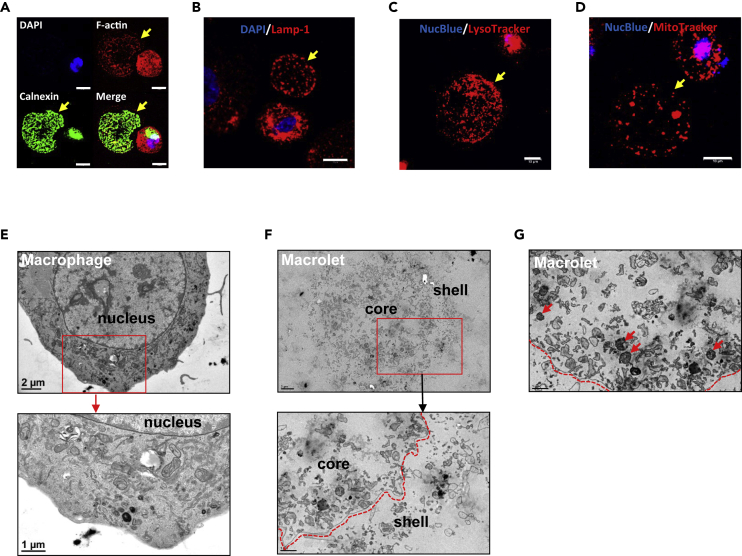
Macrolets Are Composed of Mitochondria, Lysosomes, and ER Structures (A) Representative immunofluorescent images show that macrolet (arrow) stained with Alexa Fluor 594-phalloidin (red) is enriched in calnexin (green); >10 images were collected. Scale bar, 10 μm. Note the expected perinuclear staining pattern of calnexin in the macrophage. (B) Representative image shows that macrolet (arrow) is positive for Lamp-1 protein (red); >10 images were collected; scale bar, 10 μm. Note the expected punctate staining pattern of Lamp-1 in the macrophage. (C) Representative image shows that macrolet (arrow) accumulates LysoTracker Red (red); >10 images were collected; scale bar, 10 μm. (D) Representative image shows that macrolet (arrow) stains positive for MitoTracker Deep Red; >10 images were collected. Scale bar, 10 μm. Note the expected staining pattern of mitochondria in the macrophage. (E) Representative transmission electron microscopic (TEM) images show the ultrastructure of a macrophage, surrounded by a lipid bilayer membrane and containing a nucleus and numerous intracellular organelles. Scale bar, 1 or 2 μm. (F) Representative TEM images indicate that macrolets are composed of an interior "core" surrounded by a single membrane (dotted line) and an outer “shell”. Scale bar, 1 or 2 μm. (G) Representative TEM images show that macrolets contain a variety of intracellular organelles including mitochondria (arrow), vesicles, and autophagic vacuoles. Scale bar, 1 μm.

Considering their large size and structure, we asked whether macrolets contain subcellular organelles such as endoplasmic reticulum (ER), lysosomes, and mitochondria. In immunofluorescence studies (Figure 2A), calnexin, a principal protein-folding chaperone in the ER, was localized proximal to the nucleus in LPS-stimulated THP-1 cells; however, macrolets stained diffusely for calnexin, in contrast to exosomes that do not contain calnexin (Wen et al., 2017). Using immunofluorescent staining with the lysosomal markers Lamp-1 and the vital fluorescent reporter LysoTracker, we also detected lysosomes (Figures 2B and 2C). Presence of lysosomes was consistent with diffuse immunofluorescent staining for the vacuolar H^+^-ATPase (Figure S3), the principal proton transporter responsible for the acidification of the interior of lysosomes, autophagic vacuoles, and secretory compartments (Sun-Wada and Wada, 2013). Lastly, using the vital fluorescent reporter MitoTracker Deep Red, we found macrolets also contained mitochondria (Figure 2D). These cytological surveys provide evidence that the macrolet contains organelles associated with signature macrophage functions such as protein secretion and degradation, as well as uptake and killing of bacteria.

To confirm these observations and further explore macrolet ultrastructure, transmission electron microscopy (TEM) was performed. Profoundly different from macrophages as shown in Figures 2E and 2F, macrolets were organized into “core” and “shell” domains. Unlike the lipid bilayer membrane of the macrophage, the core of the macrolet was delimited by a single membrane encompassing the entire macrolet (Figure S4A). Moreover, mitochondria, vesicular structures, and autophagic vacuoles were also observed (Figure 2G) confirming our observations using vital reporters and immunofluorescent imaging. At high-power magnification (Figure S4B), interesting differences in mitochondrial morphology were observed showing that mitochondria located in macrophages were elongated, whereas those located in macrolets were shorter and fragmented, potentially reflecting differences in their accessibility to LPS or differential influences of LPS on metabolism (Kang et al., 1990, Kang et al., 1992).

**Figure 3:**
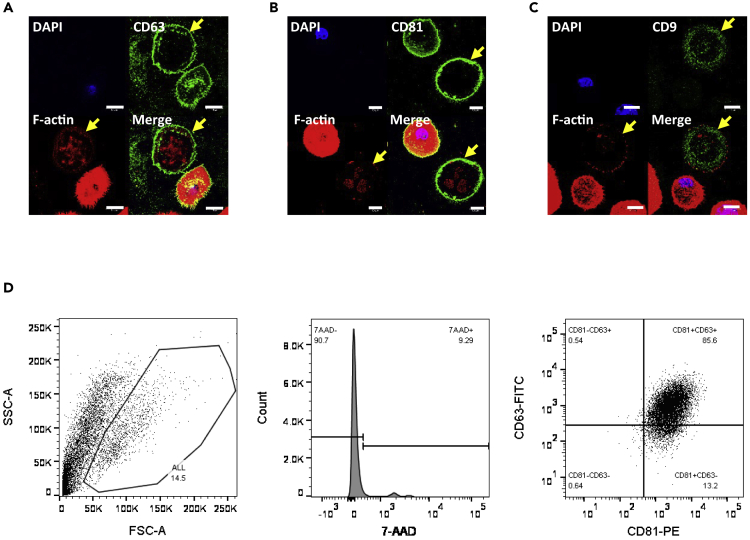
Tetraspanins Such as CD9, CD63, and CD81 Are Highly Expressed in Macrolets (A) Representative immunofluorescent images of THP-1 macrophages treated with LPS (100 ng/mL) for 4 h showed that CD63 (green) was expressed on the cell membrane of THP-1 macrophages and the surface of macrolets (arrow); Alexa Fluor 594-phalloidin (F-actin; red); scale bar, 10 μm. (B) Representative immunofluorescent images of THP-1 macrophages treated with LPS for 4 h showed that CD81 (green) is predominantly expressed on the cell membrane of THP-1 macrophages and the surface of macrolets (arrow); Alexa Fluor 594-phalloidin (F-actin; red); scale bar, 10 μm. (C) Representative immunofluorescent images of THP-1 macrophages treated with LPS for 4 h showed that CD9 (green) is not expressed on the cell membrane of THP-1 macrophages or at the surface of macrolets (arrow); Alexa Fluor 594-phalloidin (F-actin; red); scale bar, 10 μm. (D) Culture medium was collected from LPS-stimulated THP-1 macrophages (LPS 100 ng/mL, 4 h), and the medium was centrifuged at 5,500 × *g* for 25 min to pellet the insoluble fraction prior to flow cytometry assays. To analyze macrolet population, the cell debris and clumped cells were eliminated with side and forward scatters (left panel), then 7-AAD staining was used to exclude dead cell bodies that contain either intact or damaged nucleus (middle panel), and the 7-AAD^-^ macrolet population was further separated with CD81-PE and CD63-FITC channels (right panel).

To explore potentially common origins between macrolets and exosomes, we performed immunofluorescent imaging for tetraspanin proteins (Kowal et al., 2016). The tetraspanin CD63 localized predominantly at the cell membrane of the macrophage, and proximal to the outer border of the macrolet (Figure 3A). A similar staining pattern was observed for CD81 (Figure 3B); however, CD9 exhibited a relatively diffuse staining pattern within the macrolet (Figure 3C). To stratify macrolet populations based on expression levels of CD81 and CD63, we performed flow cytometry. Culture medium was collected from THP-1 macrophages after LPS stimulation and was centrifuged at 5,500 × *g* for 25 min to pellet the insoluble fraction. As shown in Figure 3D, we first eliminated cell debris and the nucleus-positive population with 7-aminoactinomycin D (7-AAD) labeling and found that 85.6% of macrolets express both CD81 and CD63, 13.2% of macrolets are CD81^+^CD63^-^, 0.6% of them only express CD63 protein, and the remaining 0.6% of macrolets express neither CD81 nor CD63.

In addition, as shown in Figure 3D, we found only a very small number of macrolets in the media bathing LPS-stimulated macrophages, whereas great numbers were adherent to the culture plates. We made trials of several methods to detach macrolets from their culture dishes (trypsin EDTA, Accutase, and Accumax, which is the concentrated Accutase). Using these methods, we were only able to obtain fragments and not enough intact macrolets for separation by density gradients or by fluorescence-activated cell sorting.

### Functional Capabilities of Macrolets: Production of Pro-inflammatory Cytokines and Responses to Live *E*. *coli*

**Figure 4:**
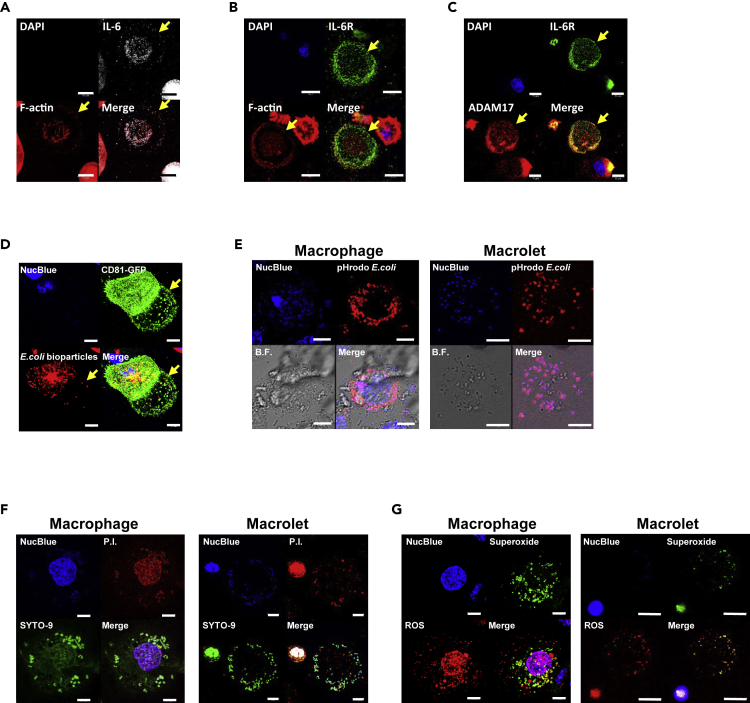
Macrolets Express both IL-6 and IL-6R and Are Able to Trap and Kill *E*. *coli* Bacteria (A) Representative immunofluorescent images show that macrolets express IL-6 (gray). Alexa Fluor 594-phalloidin (F-actin; red); scale bar, 10 μm. (B) Representative immunofluorescent images show that macrolets express IL-6R (green). Alexa Fluor 594-phalloidin (F-actin; red); scale bar, 10 μm. (C) Representative immunofluorescent images show that IL-6R (green) is profoundly co-localized with ADAM-17 (red) in macrolets. Ten macrolets were randomly picked for fluorescence intensity and co-localization analysis using Imaris 9.0 software showing Pearson's coefficient = 0.70 ± 0.09. Data were obtained and analyzed from three independent experiments. (D) Representative images show that *E*. *coli* bioparticles were captured by both CD81-GFP^+^ macrophages and macrolets (arrow); scale bar, 10 μm. (E) THP-1 macrophages were co-cultured with pH-sensitive *E*. *coli* (pHrodo) for 4 h. Confocal images were collected to assess the internalization of pHrodo *E*. *coli* in both macrophages and macrolets. Representative images show that pHrodo *E*. *coli* were taken up into acidic vacuoles in both macrophages and macrolets; scale bar, 10 μm. (F) THP-1 macrophages stimulated with LPS were co-cultured with live *E*. *coli* (strain HB101) for 4 h. The viability of *E*. *coli* bacteria was assessed using propidium iodide staining (P.I., red; dead bacteria) and compared with SYTO-9 staining (green, total bacteria); scale bar, 10 μm. About 89.7% of bacteria engulfed by macrophages (n = 20) were dead, and 69.3% of bacteria captured by macrolets (n = 20) were dead. Data were obtained and analyzed from three independent experiments. (G) THP-1 macrophages were stimulated with 100 ng/mL of LPS for 4 h, and the production of reactive oxygen species (ROS; red) and superoxide (green) was assessed using fluorescent staining. Representative images show that macrophages (left panel) and macrolets (right panel) were able to produce ROS and superoxide; scale bar, 10 μm.

As release of IL-6 and expression of the IL-6 receptor (IL-6R) are among the earliest and potentially actionable cytokine responses to trauma and infection (Namas et al., 2013, Netea et al., 2017, Prenissl et al., 2019, Zhang et al., 2014), we determined the relative expression of IL-6 and its receptor in an enriched population of macrolets. Immunofluorescent studies illustrated that both IL-6 (Figure 4A) and IL-6R (Figure 4B) were detected in macrolets sourced from LPS-stimulated THP-1 cells.

The interaction of IL-6 with its receptor has two dimensions (Garbers and Rose-John, 2018): a classic signaling pathway, which refers to interactions with IL-6R (full-length) localized at the cell membrane, and the so-called trans-signaling pathway that refers to interactions with a soluble form of IL-6R and assembly of a soluble pro-inflammation complex with the ubiquitously expressed receptor subunit gp130. Formation of the soluble form results from cleavage of the full-length form, mediated by metalloproteinases such as ADAM-10 and ADAM-17 (Garbers et al., 2011, Schumacher et al., 2015). To demonstrate the capability of macrolets to generate the components of both pathways, we used immunofluorescent staining to show that ADAM-17 was highly expressed in macrolets sourced from LPS-stimulated THP-1 cells and localized in close proximity to IL-6R (Figure 4C). The Pearson's coefficient for the colocalization between ADAM-17 and IL-6R was 0.70 ± 0.09, suggesting these two proteins were predominantly located in the same compartments within the macrolets.

Based on their robust release and cytokine signals in response to soluble LPS, we hypothesized that macrolets would harbor mechanisms for trapping microbes such as *E*. *coli*, thereby amplifying the capabilities of the stimulated macrophage. To track the behavior of macrolets, we developed a stable THP-1 cell line expressing a green fluorescent protein-tagged CD81 (CD81-GFP). As shown in Figure S5, CD81-GFP expression localized to the cell surface in macrophages and proximal to the periphery in macrolets, with patterns similar to those observed for endogenous CD81. In addition, transduction of cells with the GFP-CD81 construct did not interfere with the discoid morphology of the macrolet as shown in Video S4.

Video S4. The Discoid Morphology of the Macrolets Was Well-Maintained When THP-1 Cells Were Transduced with CD81-GFP Lentiviral Particles, Related to Figure 4 Z stack confocal microscopic video shows that a CD81-GFP + macrolet was released and its discoid morphology is well maintained with expression of a CD81-GFP construct delivered by lentiviral particles.

Using GFP-CD81 constructs as tools for visualizing macrophages and macrolets, we were able to show that macrolets are able to capture *E*. *coli* particles (K-12 strain) conjugated with Texas Red (Figure 4D). In further studies, microbes were loaded with the pH-sensitive fluorescent reporter pHrodo Red *E*.*coli* bioparticles, which turn red when bacteria are internalized in acidic compartments such as phagolysosomes (Figure 4E). We found a similar degree of pHrodo Red *E*. *coli* bioparticles activated in macrophages (88.4%) and macrolets (83.3%), illustrating the biological activity of phagocytosis in macrolets. To test whether the bacteria trapped in macrolets were vulnerable to bactericidal activity, we co-cultured LPS-stimulated macrophages with live *E*. *coli* for 4 h and then measured bacterial viability in macrophages and macrolets using propidium iodide (P.I.) to stain for dead bacteria and SYTO-9 to stain for total bacteria. As shown in Figure 4F, we found that 89.7% of bacteria were killed after engulfment by macrophages, whereas 69.3% of *E*. *coli* captured by macrolets were not viable. Also, as shown in Figure 4G, similar to macrophages, macrolets were capable of generating both reactive oxygen species (ROS) and superoxide, which in conjunction with acidification, would be the likely mechanism of bacterial killing within a phagolysosome.

### Macrolets Are Detected in Other Models of Monocyte/Macrophage Function

In a final set of studies, we found that macrolets were also detected following LPS stimulation of RAW 264.7 cells (Figure S6A), a mouse monocyte/macrophage lineage, and human primary monocytes transformed to M1 macrophages by pre-treatment with human recombinant macrophage colony stimulating factor (M-CSF) for 4 days, and then treated with LPS and interferon γ (IFN-γ) for additional 48 h (Figure S6B). From these studies it seems reasonable to infer that biogenesis of macrolets following LPS stimulation is not species specific, nor is it a property only of transformed cells.

## Discussion

Our studies provide *in vitro* evidence of a population of outsized (10–30 μm) EVs, released in response to LPS, that share attributes of smaller exosome populations but that also have capabilities attributed to a macrophage. To our knowledge, this is the first report providing details of their morphology, structure, composition, and potential range of functions. Our studies identify macrolet populations as distinct from exosomes and other EVs by size and the presence of organelles such as ER, mitochondria, and phagolysosomes. Also excluded by our studies is the possibility that they represent by-products of dying cells (Bergsbaken et al., 2009, Broz, 2015). Lastly, their morphology, delimitation by a lipid membrane, the lack of association with dying cells, and presence of organelles distinguish macrolets from extracellular traps despite the shared characteristic of microbe trapping (Brinkmann and Zychlinsky, 2012, Doster et al., 2018, Sharma et al., 2017).

The production of such "macrolets" by cells of the innate immune system has been suggested recently, through methods of separation and proteomic analysis (Kowal et al., 2016). Our studies demonstrate that macrolets are capable of producing pro-inflammatory cytokines such as IL-6 and IL-6R that have also been linked to the initial responses to infection, trauma, and shock (Garbers et al., 2011, Prenissl et al., 2019, Schumacher et al., 2015, Zhang et al., 2014). Strikingly, macrolets also express critical metalloproteins such as ADAM-17, which is critical in the processing of the IL-6R and other extracellular signals that constitute the "sheddome" (Gutierrez-Lopez et al., 2011). In addition, we find that macrolets are capable of trapping and killing bacteria, in association with well-recognized bactericidal mechanisms such as phagosomal acidification and production of ROS. Underpinning their functional capabilities, macrolets contain organelles such as ER, mitochondria, phagosomes, and lysosomes, which suggests the potential for flexibility and a kind of sustainability in response to injury or infection. These general considerations suggest that macrolets may serve as extenders and amplifiers of macrophage functions—such as production of cytokines or phagocytosis of foreign material—in injured tissue or spaces such as peritoneum or pleura in response to infection or irritation. In addition, it offers the possibility that larger EV populations such as macrolets might serve as important modifiers and therapeutic targets for disease states driven by failed resolution of innate immunity (Serhan, 2017a, Serhan, 2017b, Serhan et al., 2018).

These observations raise three additional issues for discussion. First, we have found that macrolets, although present in small numbers under baseline conditions, are released in substantial numbers following stimulation with LPS. Among macrophages, the principal mechanism for recognizing and responding to LPS is the Toll-like receptor 4 (TLR4) pathway, which plays a pivotal role in the sensing and processing danger and harmful signals known as pathogen-associated molecular patterns (PAMPs) (Jaekal et al., 2007, Lu et al., 2008, Muta and Takeshige, 2001). Based on these considerations, it is likely that the biogenesis of macrolets is mediated by the TLR4 pathway, and the underlying mechanism needs to be further elucidated. Also, it would be of great interest to know whether macrolets can be released in response to other PAMPs or danger signals and whether macrolets formed under different stimuli carry different sets of bioactive molecules (e.g., miRNA, cytokines, and chemokines) to communicate different messages to target tissues and cells.

A second issue is the role of tetraspanins in facilitating macrolet function. Tetraspanins play potentially critical roles in the adhesion of a wide variety of bacterial species to host cells (Green et al., 2011, Hassuna et al., 2017). Both anti-CD63 monoclonal antibody and knockdown of CD63 in human monocyte-derived macrophages (MDM) greatly inhibited the binding capacity between *Salmonella typhimurium* and MDM (Hassuna et al., 2017). We found that tetraspanin proteins CD63, CD81, and CD9 were enriched in macrolets, suggesting that they may serve similar roles as adhesion molecules to capture pathogenic microbes. More broadly, tetraspanins have been implicated in biogenesis of exosomes, assembly of compartments, and selection of proteins such as ADAM-17 for recruitment and stabilization at the membrane (Andreu and Yanez-Mo, 2014, Gutierrez-Lopez et al., 2011, Perez-Hernandez et al., 2013, Tsukamoto et al., 2014). Our observations indicate that therapeutic targeting of exosomes through their expression of tetraspanins (Rana et al., 2012, Rana and Zoller, 2011, Yim and Choi, 2016) may need to take into account their enrichment in larger EV populations (Kowal et al., 2016) and the potential for unintended/off-target effects.

A third issue is the mechanism by which, following release, a macrolet may be transported to its site of activity as an extender/amplifier of the functions of the source macrophage. Although we have no direct evidence for motility of macrolets too far beyond the source macrophage, it is intriguing that they have a discoid morphology. By analogy with the erythrocyte, it is tempting to speculate that such a morphology implies a high surface to volume ratio (Diez-Silva et al., 2010), which may, in turn, facilitate large reversible elastic formation as the macrolet engages with its microenvironment. Our studies provide evidence that shape is maintained by an actin cytoskeleton, suggesting that this discoid shape may not be a response only to the two-dimensional platform of a microscope slide. Further studies would be needed to explore whether shape and biomechanical characteristics are observed in three-dimensional organ culture models and how they influence function.

### Limitations of the Study

A principal limitation of this study is that most of our observations have been made in an *in vitro* system, using THP-1 macrophage-like cells exposed to a danger signal such as purified LPS. Extending these observations to more complex conditions of cell co-culture and *in vivo* models of infection and sepsis will provide more detailed information about the conditions leading to formation and release, longevity, and biological importance of this new class of EVs. At the cellular level, it will be of great interest to determine their mechanisms of biogenesis and release.

## Methods

All methods can be found in the accompanying Transparent Methods supplemental file.

## Supplemental Information

Document S1. Transparent Methods and Figures S1–S6

## References

[bib1] Akers J.C., Gonda D., Kim R., Carter B.S., Chen C.C. (2013). Biogenesis of extracellular vesicles (EV): exosomes, microvesicles, retrovirus-like vesicles, and apoptotic bodies. J. Neuro Oncol..

[bib2] Andreu Z., Yanez-Mo M. (2014). Tetraspanins in extracellular vesicle formation and function. Front. Immunol..

[bib3] Ariel A., Serhan C.N. (2012). New lives given by cell death: macrophage differentiation following their encounter with apoptotic leukocytes during the resolution of inflammation. Front. Immunol..

[bib4] Bergsbaken T., Fink S.L., Cookson B.T. (2009). Pyroptosis: host cell death and inflammation. Nat. Rev. Microbiol..

[bib5] Bhatnagar S., Shinagawa K., Castellino F.J., Schorey J.S. (2007). Exosomes released from macrophages infected with intracellular pathogens stimulate a proinflammatory response in vitro and in vivo. Blood.

[bib6] Brinkmann V., Zychlinsky A. (2012). Neutrophil extracellular traps: is immunity the second function of chromatin?. J. Cell Biol..

[bib7] Broz P. (2015). Immunology: caspase target drives pyroptosis. Nature.

[bib8] Cullen S.P., Kearney C.J., Clancy D.M., Martin S.J. (2015). Diverse activators of the NLRP3 inflammasome promote IL-1beta secretion by triggering necrosis. Cell Rep..

[bib9] Dalli J., Serhan C.N. (2017). Pro-Resolving mediators in regulating and conferring macrophage function. Front. Immunol..

[bib10] Diez-Silva M., Dao M., Han J., Lim C.T., Suresh S. (2010). Shape and biomechanical characteristics of human red blood cells in health and disease. MRS Bull..

[bib11] Doster R.S., Rogers L.M., Gaddy J.A., Aronoff D.M. (2018). Macrophage extracellular traps: a scoping review. J. Innate Immun..

[bib12] Dreyer F., Baur A. (2016). Biogenesis and functions of exosomes and extracellular vesicles. Methods Mol. Biol..

[bib13] Esser J., Gehrmann U., D'Alexandri F.L., Hidalgo-Estevez A.M., Wheelock C.E., Scheynius A., Gabrielsson S., Radmark O. (2010). Exosomes from human macrophages and dendritic cells contain enzymes for leukotriene biosynthesis and promote granulocyte migration. J. Allergy Clin. Immunol..

[bib14] Fitzner D., Schnaars M., van Rossum D., Krishnamoorthy G., Dibaj P., Bakhti M., Regen T., Hanisch U.K., Simons M. (2011). Selective transfer of exosomes from oligodendrocytes to microglia by macropinocytosis. J. Cell Sci..

[bib15] Garbers C., Janner N., Chalaris A., Moss M.L., Floss D.M., Meyer D., Koch-Nolte F., Rose-John S., Scheller J. (2011). Species specificity of ADAM10 and ADAM17 proteins in interleukin-6 (IL-6) trans-signaling and novel role of ADAM10 in inducible IL-6 receptor shedding. J. Biol. Chem..

[bib16] Garbers C., Rose-John S. (2018). Dissecting interleukin-6 classic- and trans-signaling in inflammation and cancer. Methods Mol. Biol..

[bib17] Garzetti L., Menon R., Finardi A., Bergami A., Sica A., Martino G., Comi G., Verderio C., Farina C., Furlan R. (2014). Activated macrophages release microvesicles containing polarized M1 or M2 mRNAs. J. Leukoc. Biol..

[bib18] Ginhoux F., Jung S. (2014). Monocytes and macrophages: developmental pathways and tissue homeostasis. Nat. Rev. Immunol..

[bib19] Green L.R., Monk P.N., Partridge L.J., Morris P., Gorringe A.R., Read R.C. (2011). Cooperative role for tetraspanins in adhesin-mediated attachment of bacterial species to human epithelial cells. Infect. Immun..

[bib20] Gutierrez-Lopez M.D., Gilsanz A., Yanez-Mo M., Ovalle S., Lafuente E.M., Dominguez C., Monk P.N., Gonzalez-Alvaro I., Sanchez-Madrid F., Cabanas C. (2011). The sheddase activity of ADAM17/TACE is regulated by the tetraspanin CD9. Cell Mol. Life Sci..

[bib21] Hassuna N.A., Monk P.N., Ali F., Read R.C., Partridge L.J. (2017). A role for the tetraspanin proteins in Salmonella infection of human macrophages. J. Infect..

[bib22] Hundertmark J., Krenkel O., Tacke F. (2018). Adapted immune responses of myeloid-derived cells in fatty liver disease. Front. Immunol..

[bib23] Ismail N., Wang Y., Dakhlallah D., Moldovan L., Agarwal K., Batte K., Shah P., Wisler J., Eubank T.D., Tridandapani S. (2013). Macrophage microvesicles induce macrophage differentiation and miR-223 transfer. Blood.

[bib24] Jaekal J., Abraham E., Azam T., Netea M.G., Dinarello C.A., Lim J.S., Yang Y., Yoon D.Y., Kim S.H. (2007). Individual LPS responsiveness depends on the variation of toll-like receptor (TLR) expression level. J. Microbiol. Biotechnol..

[bib25] Johnson S.M., Dempsey C., Parker C., Mironov A., Bradley H., Saha V. (2017). Acute lymphoblastic leukaemia cells produce large extracellular vesicles containing organelles and an active cytoskeleton. J. Extracell. Vesicles.

[bib26] Kang Y.H., Dwivedi R.S., Lee C.H. (1990). Ultrastructural and immunocytochemical study of the uptake and distribution of bacterial lipopolysaccharide in human monocytes. J. Leukoc. Biol..

[bib27] Kang Y.H., Lee C.H., Monroy R.L., Dwivedi R.S., Odeyale C., Newball H.H. (1992). Uptake, distribution and fate of bacterial lipopolysaccharides in monocytes and macrophages: an ultrastructural and functional correlation. Electron. Microsc. Rev..

[bib28] Kowal J., Arras G., Colombo M., Jouve M., Morath J.P., Primdal-Bengtson B., Dingli F., Loew D., Tkach M., Thery C. (2016). Proteomic comparison defines novel markers to characterize heterogeneous populations of extracellular vesicle subtypes. Proc. Natl. Acad. Sci. U S A.

[bib29] Lanyu Z., Feilong H. (2019). Emerging role of extracellular vesicles in lung injury and inflammation. Biomed. Pharmacother..

[bib30] Lu Y.C., Yeh W.C., Ohashi P.S. (2008). LPS/TLR4 signal transduction pathway. Cytokine.

[bib31] Muta T., Takeshige K. (2001). Essential roles of CD14 and lipopolysaccharide-binding protein for activation of toll-like receptor (TLR)2 as well as TLR4 Reconstitution of TLR2- and TLR4-activation by distinguishable ligands in LPS preparations. Eur. J. Biochem..

[bib32] Namas R.A., Bartels J., Hoffman R., Barclay D., Billiar T.R., Zamora R., Vodovotz Y. (2013). Combined in silico, in vivo, and in vitro studies shed insights into the acute inflammatory response in middle-aged mice. PLoS One.

[bib33] Netea M.G., Balkwill F., Chonchol M., Cominelli F., Donath M.Y., Giamarellos-Bourboulis E.J., Golenbock D., Gresnigt M.S., Heneka M.T., Hoffman H.M. (2017). A guiding map for inflammation. Nat. Immunol..

[bib34] O'Neill H.C., Quah B.J. (2008). Exosomes secreted by bacterially infected macrophages are proinflammatory. Sci. Signal..

[bib35] Perez-Hernandez D., Gutierrez-Vazquez C., Jorge I., Lopez-Martin S., Ursa A., Sanchez-Madrid F., Vazquez J., Yanez-Mo M. (2013). The intracellular interactome of tetraspanin-enriched microdomains reveals their function as sorting machineries toward exosomes. J. Biol. Chem..

[bib36] Pieters B.C.H., Cappariello A., van den Bosch M.H.J., van Lent P., Teti A., van de Loo F.A.J. (2019). Macrophage-derived extracellular vesicles as carriers of alarmins and their potential involvement in bone homeostasis. Front. Immunol..

[bib37] Prenissl N., Lokau J., Rose-John S., Haybaeck J., Garbers C. (2019). Therapeutic blockade of the interleukin-6 receptor (IL-6R) allows sIL-6R generation by proteolytic cleavage. Cytokine.

[bib38] Rana S., Yue S., Stadel D., Zoller M. (2012). Toward tailored exosomes: the exosomal tetraspanin web contributes to target cell selection. Int. J. Biochem. Cell Biol..

[bib39] Rana S., Zoller M. (2011). Exosome target cell selection and the importance of exosomal tetraspanins: a hypothesis. Biochem. Soc. Trans..

[bib40] Schumacher N., Meyer D., Mauermann A., von der Heyde J., Wolf J., Schwarz J., Knittler K., Murphy G., Michalek M., Garbers C. (2015). Shedding of endogenous interleukin-6 receptor (IL-6R) is governed by A disintegrin and metalloproteinase (ADAM) proteases while a full-length IL-6R isoform localizes to circulating microvesicles. J. Biol. Chem..

[bib41] Serhan C.N. (2017). Discovery of specialized pro-resolving mediators marks the dawn of resolution physiology and pharmacology. Mol. Aspects Med..

[bib42] Serhan C.N. (2017). Treating inflammation and infection in the 21st century: new hints from decoding resolution mediators and mechanisms. FASEB J..

[bib43] Serhan C.N., Chiang N., Dalli J. (2018). New pro-resolving n-3 mediators bridge resolution of infectious inflammation to tissue regeneration. Mol. Aspects Med..

[bib44] Sharma R., O’Sullivan K.M., Holdsworth S.R., Bardin P.G., King P.T. (2017). Visualizing macrophage extracellular traps using confocal microscopy. J. Vis. Exp..

[bib45] Sun-Wada G.H., Wada Y. (2013). Vacuolar-type proton pump ATPases: acidification and pathological relationships. Histol. Histopathol..

[bib46] Thery C., Witwer K.W., Aikawa E., Alcaraz M.J., Anderson J.D., Andriantsitohaina R., Antoniou A., Arab T., Archer F., Atkin-Smith G.K. (2018). Minimal information for studies of extracellular vesicles 2018 (MISEV2018): a position statement of the International Society for Extracellular Vesicles and update of the MISEV2014 guidelines. J. Extracell Vesicles.

[bib47] Tomlin H., Piccinini A.M. (2018). A complex interplay between the extracellular matrix and the innate immune response to microbial pathogens. Immunology.

[bib48] Tsukamoto S., Takeuchi M., Kawaguchi T., Togasaki E., Yamazaki A., Sugita Y., Muto T., Sakai S., Takeda Y., Ohwada C. (2014). Tetraspanin CD9 modulates ADAM17-mediated shedding of LR11 in leukocytes. Exp. Mol. Med..

[bib49] Vagner T., Spinelli C., Minciacchi V.R., Balaj L., Zandian M., Conley A., Zijlstra A., Freeman M.R., Demichelis F., De S. (2018). Large extracellular vesicles carry most of the tumour DNA circulating in prostate cancer patient plasma. J. Extracellular Vesicles.

[bib50] Varol C., Mildner A., Jung S. (2015). Macrophages: development and tissue specialization. Annu. Rev. Immunol..

[bib51] Vemula P.K., Kohler J.E., Blass A., Williams M., Xu C., Chen L., Jadhav S.R., John G., Soybel D.I., Karp J.M. (2014). Self-assembled hydrogel fibers for sensing the multi-compartment intracellular milieu. Sci. Rep..

[bib52] Wen C., Seeger R.C., Fabbri M., Wang L., Wayne A.S., Jong A.Y. (2017). Biological roles and potential applications of immune cell-derived extracellular vesicles. J. Extracellular Vesicles.

[bib53] Wight T.N., Frevert C.W., Debley J.S., Reeves S.R., Parks W.C., Ziegler S.F. (2017). Interplay of extracellular matrix and leukocytes in lung inflammation. Cell Immunol..

[bib54] Wynn T.A., Barron L. (2010). Macrophages: master regulators of inflammation and fibrosis. Semin. Liver Dis..

[bib55] Yim N., Choi C. (2016). Extracellular vesicles as novel carriers for therapeutic molecules. BMB Rep..

[bib56] Zhang Y., Zhang J., Korff S., Ayoob F., Vodovotz Y., Billiar T.R. (2014). Delayed neutralization of interleukin 6 reduces organ injury, selectively suppresses inflammatory mediator, and partially normalizes immune dysfunction following trauma and hemorrhagic shock. Shock.

[bib57] Zhu Z., Zhang D., Lee H., Menon A.A., Wu J., Hu K., Jin Y. (2017). Macrophage-derived apoptotic bodies promote the proliferation of the recipient cells via shuttling microRNA-221/222. J. Leukoc. Biol..

[bib58] Zhuang J., Dinsdale D., Cohen G.M. (1998). Apoptosis, in human monocytic THP.1 cells, results in the release of cytochrome c from mitochondria prior to their ultracondensation, formation of outer membrane discontinuities and reduction in inner membrane potential. Cell Death Differ..

