# Supplementary material for: Macrolets: Outsized Extracellular Vesicles Released from Lipopolysaccharide-Stimulated Macrophages that Trap and Kill Escherichia coli
Source: iScience. 2020 May 5;23(6):101135. doi: 10.1016/j.isci.2020.101135 (PMC7240733; doi:10.1016/j.isci.2020.101135)
Supplement: Document S1. Transparent Methods and Figures S1–S6 [file mmc1.pdf]

iScience, Volume *23*

Supplemental Information

Macrolets: Outsized Extracellular Vesicles Released from Lipopolysaccharide-Stimulated Macrophages that Trap and Kill *Escherichia coli*

Wei Ding, Olivia C. Rivera, Shannon L. Kelleher, and David I. Soybel

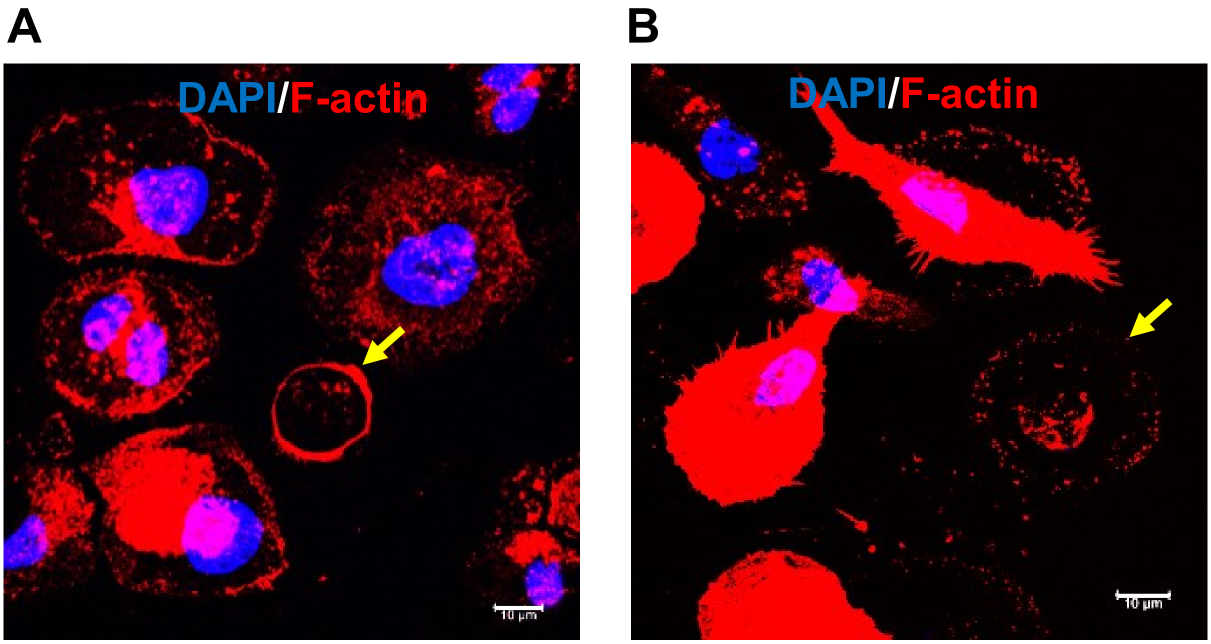

Figure S1. Related to Figure 1

Macrolets are outsized heterogeneous extracellular vesicles. (**A**) THP-1 macrophages were treated with 100 ng/ml of LPS for 4 h, and macrophages and macrolets were fixed with 4% formaldehyde and stained with Alexa Fluor-594 phalloidin (F-actin; red) and DAPI (blue). The large proportion of macrolets have a well-organized structure with relatively sparse interior F-actin staining. (**B**) A small proportion of macrolets have an outer “shell” and an interior “core” with somewhat fragmented F-actin staining (arrow). Multiple images were collected (n>10), scale bar = 10 µm.

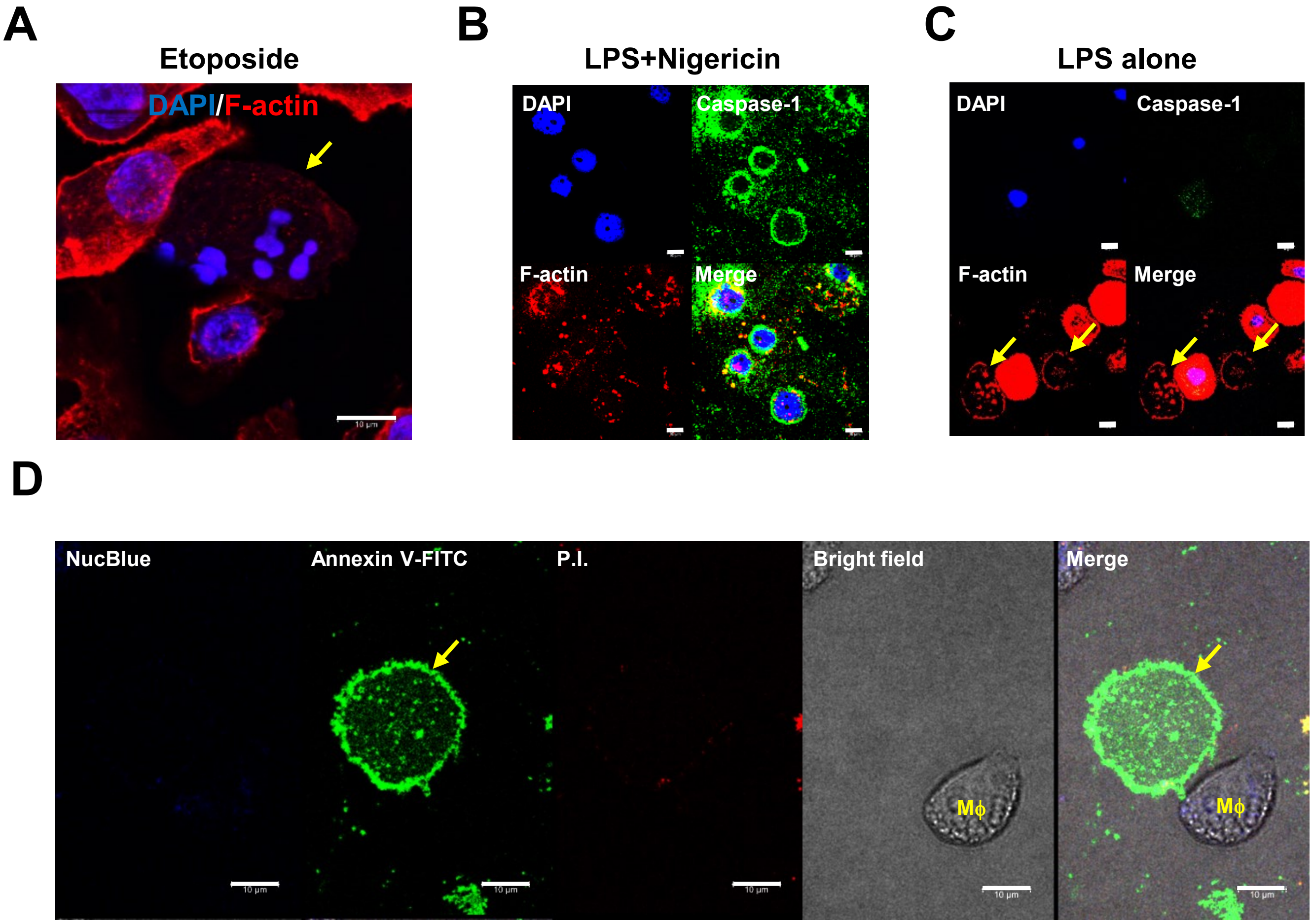

Figure S2. Related to Figure 1

Macrolets were not products from apoptosis or pyroptosis. (**A**) Apoptosis was induced with Etoposide (50 μM) for 4 h, and apoptotic cells were stained with phalloidin (F-actin; red) and DAPI (nuclear DNA; blue). A typical apoptotic cell (arrow) was characterized by morphological changes such as nuclear DNA fragmentation and cytoskeleton destruction, scale bar = 10 μm. (**B**) Pyroptosis was induced with 100 ng/ml LPS for 3 h, followed by 10 μM Nigericin for 1 h. Cells were fixed with 4% formaldehyde and then stained with caspase-1 (green), phalloidin (red) and DAPI (blue). Pyroptotic cells were characterized by cell body enlargement, cell membrane rupture, and cytoskeleton destruction. Multiple images (n=5) were collected, scale bar = 10 μm. (**C**) Cell integrity was well-maintained and macrolets were generated when THP-1 macrophages were treated with LPS alone. Multiple images (n=5) were collected, scale bar = 10 μm. (**D**) Annexin V$^+$ macrolet (arrow) was released from a viable parent macrophage (Mö) with both Annexin V and PI negative labels. Multiple images (n=5) were collected, scale bar = 10 μm.

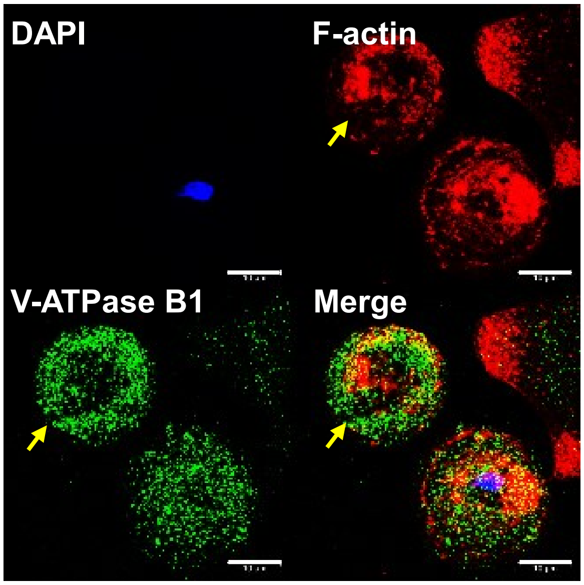

Figure S3. Related to Figure 2

Macrolets contain vacuolar H$^+$-ATPase. A representative image shows that both macrophages and macrolets (phalloidin; red, arrow) have vacuolar H$^+$-ATPase (V-ATPase B1; green). Multiple images (n=5) were collected. Scale bar = 10 μm.

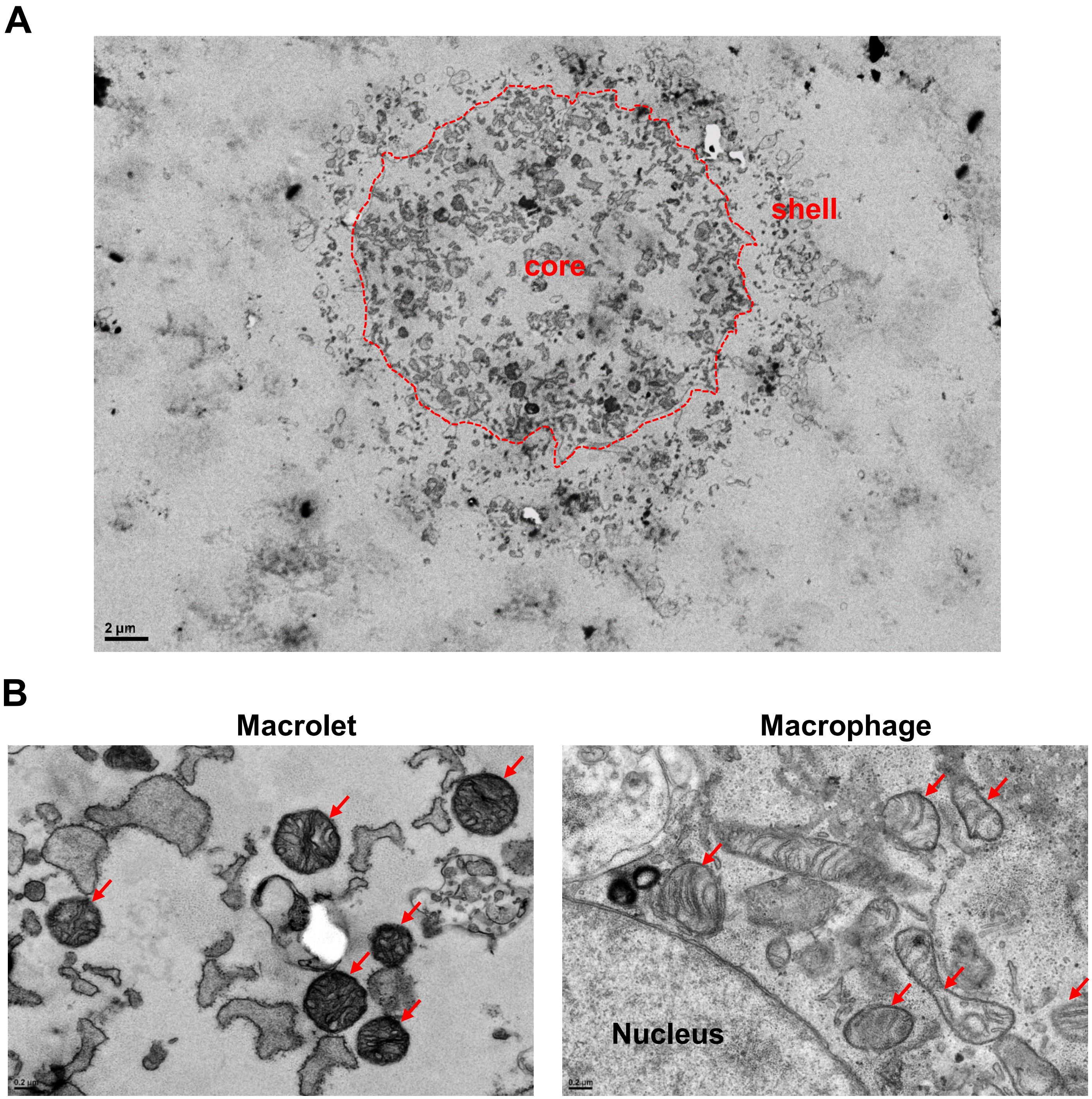

Figure S4. Related to Figure 2

(**A**) **Macrolets contain an interior core that is delimited by a single membrane.** Transmission electron microscopic (TEM) image shows that a macrolet is composed of a central core structure containing a variety of organelles and is delimited by a single membrane (highlighted with a dotted red line). Scale bar = 2 µm. (**B**) **Macrolets contain shorter fragmented mitochondria instead of elongated ones carried by macrophages.** Transmission electron microscopic (TEM) images show that mitochondria (arrow) are present in macrolets and are fragmented (left side image), which is distinct from the elongated ones that are carried by macrophages (right side image), scale bar = 0.2 µm.

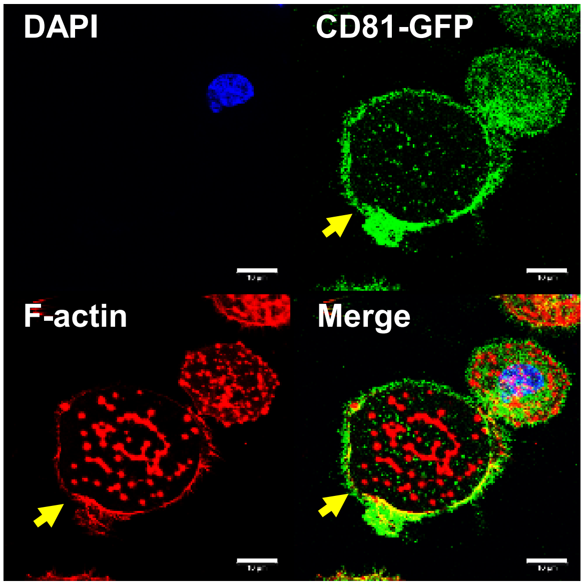

Figure S5. Related to Figure 4

Lentiviral overexpression of CD81-GFP did not interfere with the discoid morphology of the macrolets. CD81-GFP was stably expressed in THP-1 macrophages by the lentiviral transduction system. CD81-GFP expression is localized to the cell membrane of macrophages and exhibits the same expression pattern as endogenous CD81 protein as shown in Figure 3B. Scale bar = 10 μm.

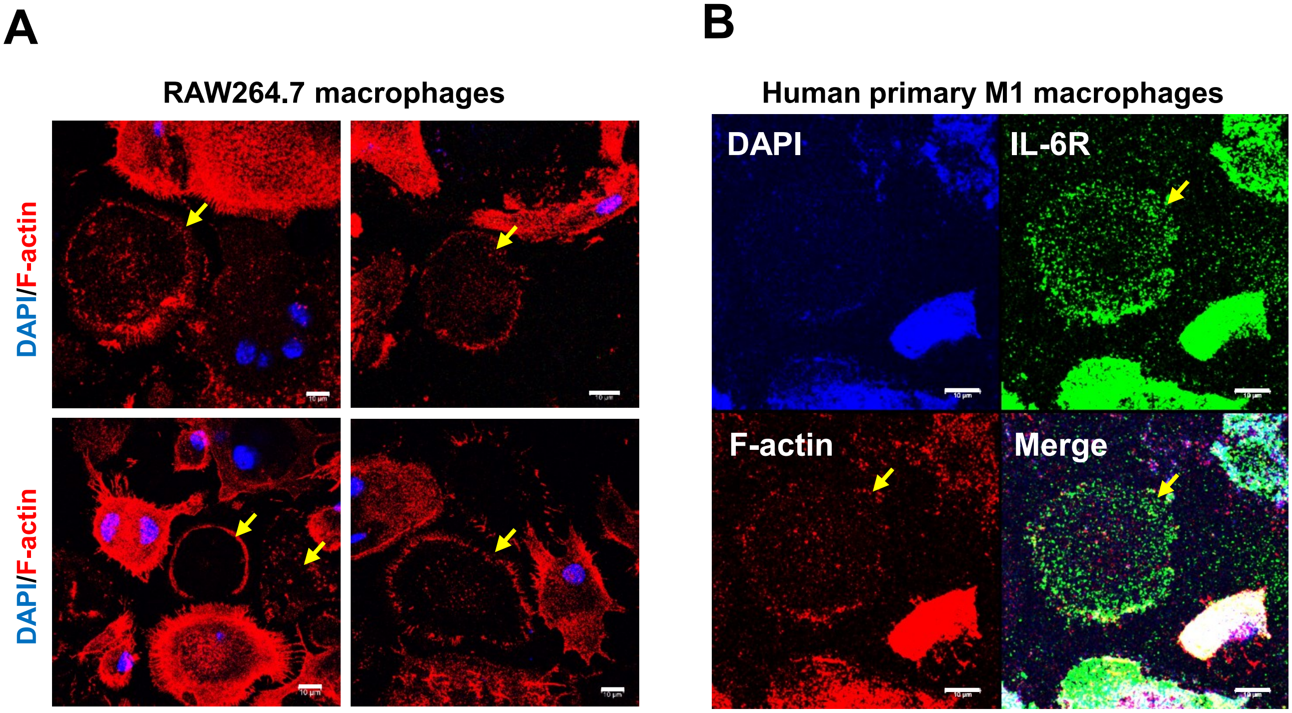

Figures S6. Related to Figure 1

Mouse and human macrophages are capable of producing macrolets. (**A**) Mouse RAW 264.7 macrophages were treated with 100 ng/ml of LPS for 4 h, and cells were fixed with 4% formaldehyde and stained with Alexa Fluor 594 phalloidin (red) and DAPI (blue). Representative images show that multiple macrolets (arrow) were released upon LPS treatment, and F-actin$^+$ macrolets are anuclear particles (DAPI$^-$). (**B**) Human primary CD14$^+$ monocytes were differentiated into M1 macrophages. Representative images show that IL-6R (green) is expressed in F-actin$^+$ macrolets (phalloidin; red) released from human primary M1 macrophages. Multiple images (n>5) were collected. Scale bar = 10 μm.

TRANSPARENT METHODS

Cells and cell culture

Human monocytic leukemia THP-1 cells were purchased from ATCC (TIB-202), and maintained in RPMI 1640 medium supplemented with 10% heat-inactivated fetal bovine serum containing 1% penicillin/streptomycin, 1×GlutaMax-I, 10 mM HEPES (pH 7.4), and 1× MEM-Non essential amino acids NEAA (Gibco). Mouse macrophage-like RAW264.7 cells were purchased from ATCC (TIB-71), and maintained in the same culture medium as for THP-1 cells. Human $CD14^+$ primary monocytes (70035) were purchased from StemCell technology (Vancouver, Canada) and differentiated into M1 macrophages using a 6-day standard protocol as recommended by the manufacturer. In brief, primary monocytes were cultured in serum-free macrophage medium (StemCell, 10961) and plated in 24-well plates ($5×10^5$ cells/well), then treated with 50 ng/ml of human recombinant macrophage colony-stimulating factor (M-CSF) for 4 days. The medium was replaced with fresh macrophage culture medium containing 10 ng/ml lipopolysaccharide (LPS, *Escherichia coli* O127:B8, Sigma-Aldrich, L5024) and 50 ng/ml IFN-γ for additional 2 days to differentiate monocytes into M1 macrophages.

Time-lapse image collection

Time-lapse images were collected using DeltaVision microscopy. In brief, THP-1 monocytes were placed in a 35-mm bottom slide dish (MetTek, P35G-0-20-C), and were differentiated with 50 ng/ml of phorbol 12-myristate 13-acetate (PMA, Sigma-Aldrich, P8139) for 48 h. The culture medium was replaced with pre-warmed Opti-MEM I supplemented with 10% FBS, and 100 ng/ml of LPS was added. Images were captured at 3-min intervals for total 4.5 h. The video was created using Volocity software.

Transmission electron microscopy

THP-1 cells ($5×10^5$ cells/well) were cultured in a MatTek bottom glass slide (MetTek, P35G-1.5-14-C-GRID), then differentiated with PMA and stimulated with 100 ng/ml of LPS for 4 h. Samples were fixed by 2.5% glutaraldehyde and 2% paraformaldehyde in 0.1 M cacodylate buffer (pH 7.4) and further fixed in 1% osmium tetroxide in 0.1 M cacodylate buffer (pH 7.4) for 1 h. Samples were dehydrated in a graduated ethanol series, and embedded in LX112 (Ladd Research, Williston, VT). Thin sections (70 nm) were stained with uranyl acetate and lead citrate and images were captured using a JEOL JEM1400 Transmission Electron Microscope (JEOL USA Inc., Peabody, MA, USA).

Immunofluorescence staining and confocal microscopy

THP-1 cells ($5×10^4$ cells/well) were cultured in 24-well plates containing a cover glass (Fisher Scientific, 12-545-83). After indicated treatments, cells were fixed in 4% methanol-free formaldehyde (Thermo Scientific Pierce, 28908) prepared in 1×PBS for 15 min. Acontaining lentiviral particles was collected and filtered with low-protein binding endotoxin-free 0.45 µm PES filters. Lentiviral particles were concentrated with the Lenti-X concentrator kit (Takara, 631231). THP-1 monocytes ($1×10^7$ cells) were transduced with 20 µl of concentrated lentivirus in 2 ml culture medium containing 8 µg/ml of polybrene for 20 min at room temperature. The spinoculation protocol was used by centrifuging cell and virus mixture for 30 min at 800×*g* at 32°C. 48 h after lentiviral transduction, 0.5 µg/ml of puromycin was added for selecting CD81-GFP positive cell pools.

MitoTracker, LysoTracker, and DiO plasma membrane staining

Mitochondria and lysosomes were stained with 500 nM of MitoTracker Deep Red FM and 75 nM of LysoTracker Red DND-99 (ThermoFisher Scientific, L7528) following the manufacturer's recommendation. Vybrant DiO cell-labeling solution (ThermoFisher Scientific, V22886) was used to stain membrane lipids. In brief, 5×10^5 cells were seeded on 35-mm Bottom Slide Dishes, and after the indicated treatment cells were stained with DiO solution diluted in Opti-MEM medium for 15 min. Dishes were washed with 1×PBS 3 times, and the confocal images were collected using a Leica TCS SP8 laser scanning fluorescence microscope.

Flow cytometry

THP-1 monocytes were differentiated to macrophages with 50 ng/ml of PMA for 48 h, and then treated with 100 ng/ml of LPS for 4 h to induce macrolet production. Culture medium was centrifuged at 5,500 × g for 25 min to pellet the insoluble fraction. Pellets were washed with 1×PBS and re-suspended in 100 µl of flow cytometry buffer (1×PBS, 2% FBS, 0.09% sodium azide), and then labeled with CD81-PE (BioLegend, Cat#349505, clone 5A6, 1:100 dilution), CD63-FITC (BioLegend, Cat#363006, clone H5C6, 1:100 dilution), and 1 µl of 7-aminoactinomycin D (7-AAD, ThermoFisher Scientific, Cat#A1310, 1 mg/ml) on ice for additional 20 min in dark. Flow cytometry was performed using BD LSRFortessa and data were analyzed with FlowJo software (BD Biosciences).

Bacteria trapping and killing assays

For bacteria trapping assay, THP-1 cells (5×10^5 cells/well) expressing CD81-GFP were plated on glass-bottom slides. Release of macrolets was induced with 100 ng/ml of LPS for 1 h, then *E.coli* (K-12 strain) bioparticles conjugated with Texas Red (ThermoFisher Scientific, Cat$^{\#}$ E2863) were added to the culture medium for additional 3 h followed by Hoechst 33324 (NucBlue) nuclear staining for 15 min. The bacteria trapping was visualized with Leica confocal microscopy. For bacterial killing assays, THP-1 cells were cultured in antibiotic-free culture medium for three passages to wash away the residual antibiotics. THP-1 monocytes (5×10^5 cells/well) were plated in 35-mm bottom slide dish, and differentiated into macrophages with 50 ng/ml of PMA for 48 h, then 5 µl of live *E.coli* bacteria (strain$^{\#}$ HB101; OD 670 nm of 0.03) were incubated with LPS-stimulated THP-1 macrophages for 4 h, and bacterial killing capability was assessed using Live/Dead BacLight Bacterial Viability Kit with SYTO-9 and propidium iodide dyes (ThermoFisher Scientific, Cat$^{\#}$ L7012).

Statistical analysis

Data presented are mean ± SD or SE as indicated in figure legends. The number for each experiment is noted in the figure legends. Student's *t* test was used for statistical analysis, and a significant difference was determined at p value < 0.05.
